# MXenes Surface Termination under Photoexcitation: Insights from Excited-State Pourbaix Diagrams

**DOI:** 10.1021/acsami.6c00715

**Published:** 2026-03-10

**Authors:** Diego Ontiveros, Francesc Viñes, Carmen Sousa

**Affiliations:** Departament de Ciència de Materials i Química Física & Institut de Química Teòrica i Computacional (IQTCUB), 223309 Universitat de Barcelona, c/Martí i Franquès 1-11, Barcelona 08028, Spain

**Keywords:** MXenes, photocatalysis, excited state, pourbaix diagrams, density functional theory

## Abstract

MXenes have emerged as promising materials for photocatalytic hydrogen production, yet their performance is critically dependent on the specific nature of their surface terminations. While Pourbaix diagrams are routinely used to map surface stability under a certain pH and applied external potential (*U*), they traditionally neglect the influence of photoexcitation on thermodynamic preference. Here, we construct the singlet (S_0_) ground state and the lowest triplet (T_1_) excited state Pourbaix diagrams for Sc_2_C, Y_2_C, and Zr_2_C MXenes, which have previously shown promising photoactive properties, to assess how photoexcitation alters surface stability. Our results show that constant photoexcitation can significantly reshape the Pourbaix diagrams, altering the thermodynamically preferred surface terminations and thereby influencing photocatalytic behavior. Across all studied systems, terminations associated with aqueous acidic etching environments (−F, −O, −OH, −H) dominate the stability regions. For Zr_2_C, this is advantageous since −O termination is both the most stable and photoactive configuration. In contrast, for Sc_2_C and Y_2_C, the potentially more active halide and chalcogen terminations are overshadowed by aqueous- and HF-derived groups, suggesting that alternative synthesis routes will be required to stabilize the most photocatalytically favorable terminations.

## Graphical abstract

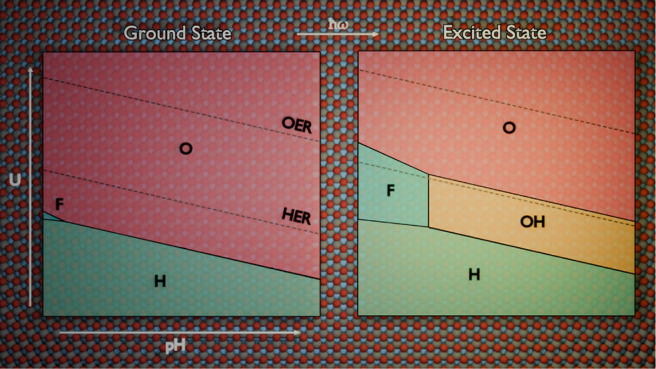

## 1 Introduction

In the early 20th century, Plotnikow (1910)^1^ and Landau (1912)^2^ first proposed the idea of carrying out a photochemical process in the presence of a compound that could catalyze the reaction with light. However, it was not until 1972 that the field experienced a true breakthrough, when Fujishima and Honda demonstrated the water-splitting capabilities of titanium dioxide (TiO_2_) photoassisted by ultraviolet (UV) light.^3^ Such paramount work completely transformed the landscape of photocatalysis, inspiring decades of intensive research and technological developments.^4^ Both homogeneous and heterogeneous systems are applied in photocatalysis nowadays, with photocatalysts ranging in dimensionality, from zero-dimensional (0D) nanoparticles to intricate two- (2D) and three-dimensional (3D) structures.

Modern photocatalysis is essential in addressing some of the world’s most pressing challenges. For example, photocatalysts can drive key chemical processes such as pollutant degradation, air and water purification, and even renewable energy generation.^5–7^ Just like the Honda-Fujishima experiment demonstrated, a well-designed photocatalyst can split water (H_2_O) to produce hydrogen (H_2_) and oxygen (O_2_) using sunlight, a sustainable and renewable energy source.^8^ The obtained H_2_ has the potential to serve as the fuel of the future, offering an appealing alternative to fossil fuels while contributing to the global transition toward cleaner energy systems.^9^ Occasionally, photocatalysis is combined with electrochemical methods, in photoelectrocatalysis or photoassisted electrocatalysis, as in the original Honda-Fujishima experiment. This involves the fixation of the photocatalyst onto a conductive substrate, which is also used as an electrode. The applied external potential thus helps separating the electron–hole pairs while preventing their recombination, boosting the overall performance.^10,11^

One currently promising class of materials for the generation of H_2_ through photocatalytic water splitting is MXenes.^12^ These 2D materials have attracted significant attention due to their growing number of applications across various fields: From energetics, where they excel as batteries and supercapacitors;^13,14^ electronics, where they show great potential as highly conductive materials or antennas;^15,16^ up to their use as sensors for different molecules.^17^ Beyond these applications, MXenes are also emerging as highly effective electro- and photocatalysts.^18,19^ They have been extensively studied for a wide range of catalytic reactions aimed at addressing the most pressing environmental challenges, such as CO_2_ capture and its electroreduction,^20,21^ the hydrogen evolution reaction (HER),^22^ and also to photocatalyze nitrogen (N_2_) fixation and the water splitting process.^23,24^

MXenes are 2D transition metal (TM) carbides and/or nitrides with M_*n*+1_X_*n*_ general chemical formula, where usually *n* = 1–4, M stands for an early TM from groups III to VI, and X can be carbon (C) and/or nitrogen (N).^25,26^ Depending on their synthesis route and chemical environment, MXenes can have their surface functionalized with a termination, T_*x*_, thus updating the chemical formula to M_*n*+1_X_*n*_T_*x*._ The common synthesis of MXenes involves selectively etching A elements (generally of groups XIII–XVI) from bulk MAX precursor materials, M_*n*+1_AX_*n*_, using aqueous hydrofluoric acid (HF),^27^ which produces terminations such as −O, −F, −OH, and occasionally −H under specific acidic and reducing conditions.^22,28^ Nevertheless, recent studies employing molten salts have reported new MXenes terminated with −S, −Se, −Te, −NH, −Cl, −Br, −I, and even pristine MXenes. Additionally, there are records of defunctionalization protocols,^29^ resulting in a large family that surpasses thousands of compounds.^30,31^

The surface termination can largely affect the performance of the MXene as a photocatalyst,^32^ as known to do on heterogeneous catalysis^33,34^ and electrocatalysis.^22,35^ In fact, pristine MXenes appear to be metallic, and only when terminated some MXenes become semiconductors.^32^ Therefore, determining the most stable termination under well-defined experimental conditions becomes key and fundamental to tune the catalyst. In photoelectrocatalysis, the reactions take place under the combined influence of light irradiation and an applied external potential, *U*, provided by a power source. Moreover, the process usually occurs in solution at a certain pH, which can also influence the surface termination stability. Within this context, Pourbaix diagrams have risen as one of the best options to study the surface stability as a function of *U* and pH experimental conditions.^36^ These diagrams have been extensively used to study the corrosion behavior, oxidation states, and surface stability of numerous systems,^37^ including MXenes.^22,38,39^ The Pourbaix diagrams can be obtained computationally through theoretical calculations, such as those gained by density functional theory (DFT).^40^

Normally, Pourbaix diagrams are computed on the electronic ground state of the system, but, when involving photocatalytic processes, the situation becomes more complex because under light irradiation the material gets excited to a higher-energy electronic state. This excited state may significantly affect the stability of the material and even change its surface chemical composition when compared to the ground state. Consequently, the surface termination that is thermodynamically stable—and actively participating in the reaction— during photocatalysis may not be the one predicted by standard ground-state models. Relying solely on S_0_ Pourbaix diagrams could lead to inaccurate predictions of a material’s photocatalytic viability. To our knowledge, no studies to date have addressed Pourbaix diagrams that explicitly consider the material’s excited state. By incorporating excited-state conditions into these stability diagrams, a more realistic understanding of how photocatalysts behave under operational conditions is attainable, ultimately helping to design more robust and efficient photocatalysts.

In this work, we develop the theoretical framework to achieve Pourbaix diagrams in the ground and excited states for candidate Sc_2_C, Y_2_C and Zr_2_C MXenes, which, when terminated, have shown favorable electronic, optical, and photocatalytic properties for water splitting under sunlight.^24^ To construct the diagrams, primary surface terminations typically produced through conventional HF-based etching methods (−F, −O, −OH, and −H) have been considered to serve as a proof of concept, although the methodology is extendable to mixed termination situations. Furthermore, for Sc- and Y-based MXenes, other possible terminations, including chalcogen (−S, −Se) and halogen (−Cl, −Br, −I) terminations, were also explored. These terminations are attainable *via* molten salt etching procedures^30^ and have been reported as promising for photocatalytic applications;^24^ however, their stability must be tested against other possible terminations arising from solution species or synthesis route.

In particular, we show here that terminations resulting from HF synthesis and aqueous solution species (−F, −O, −OH, and −H) dominate under most conditions, limiting access to halogen- and chalcogen-terminated surfaces that may be more photoactive in the cases of Sc_2_C and Y_2_C. In contrast, Zr_2_C retains −O termination as the most stable configuration under both HER and oxygen evolution reaction (OER) conditions, reinforcing its potential as a robust photocatalyst for water splitting. These results emphasize that both the excited-state environment and the synthesis route play a decisive role in determining MXene surface chemistry, providing a framework to design and control terminations under realistic photoelectrochemical conditions.

## 2 Computational Details

### 2.1 Structural Models

MXenes were modeled employing slab models within periodic boundary conditions, with 20 Å of vacuum perpendicular to the MXene surface to ensure negligible self-interaction. The minimal *p*(1 × 1) hexagonal unit cell was used, thus considering a full coverage of the explored terminations, as shown to be adequate to provide accurate results in previous computational studies on MXenes in the photocatalytic water splitting.^24,32^ As in these previous studies, given the layered nature of MXenes, two possible stacking configurations can arise for these systems: ABC stacking, see Figure 1a, where the M layers occupy two different relative positions, or ABA stacking, see Figure 1b, in which the M layers are aligned in the same relative position along the vacuum direction. Such different stackings are known to affect the electronic structure and, eventually, the surface chemical activity of MXenes, and so need to be accounted for.^33,41^ When terminated, the T_*x*_ atoms tend to occupy different surface hollow sites, and so here three for each stacking were explored, see Figure 1c. In ABC stacking, there is the metal hollow site (H_M_), located directly above an underlying M atom. In ABA stacking, the simple hollow site (H) is present, which is positioned with no atom directly underneath it. Additionally, both stackings have carbon or nitrogen hollow sites (H_X_), where the termination sits above an underlying X atom. A mixed configuration is also possible, with a combination of H_M_ (or H) and H_X_ on opposite MXene surfaces for ABC (or ABA) stacking, referred to as H_MX_, see Figure 1c.

**Figure 1:**
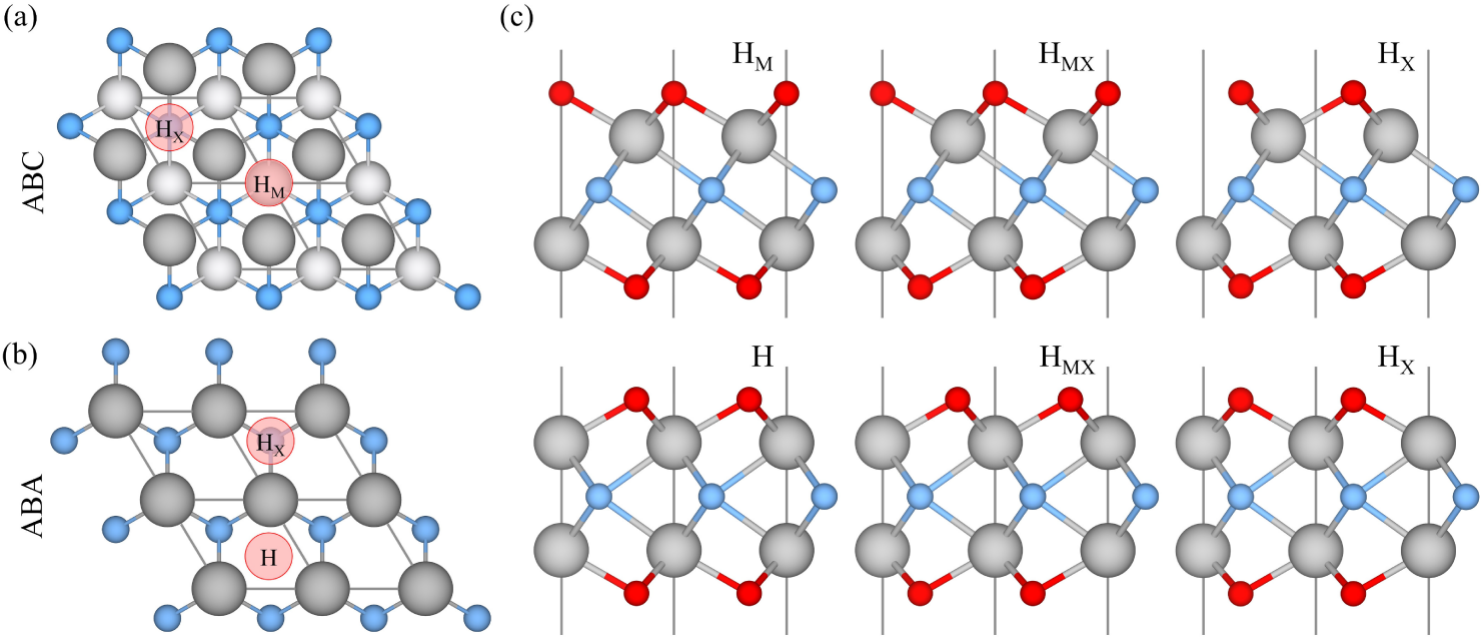
Top view of the (a) ABC and (b) ABA stacking of pristine MXenes, with the red circles marking high-symmetry points where the termination could be placed. (c) Side view of the six possible terminated MXene geometries, three for each stacking. Solid gray, blue, and red spheres represent the metal, M, X atom (C or N), and the termination atoms, T*_x_*, respectively. The thin gray lines display the limits of the unit cell.

Previous works have identified group III and group IV terminated MXenes as promising candidates for water splitting photocatalysis.^24,42,43^ More precisely, Zr_2_CO_2_, plus Sc_2_CT_2_ and Y_2_CT_2_, with T_*x*_ = −Cl, −Br, −I, −S, and −Se, present themselves as the most optimal cases.^32,44^ These systems exhibit suitable bandgaps, band alignment, and optical absorption for the water splitting reaction.^24^ Notably, in the cases of Sc_2_CT_2_ and Y_2_CT_2_, the halide terminations presenting significantly better optical absorption profiles and enhanced electron–hole separation compared to the chalcogen ones.^24^ Based on these findings, we selected these systems to study their surface nature and stability under the experimental conditions in which the photoelectrocatalytic process takes place. Besides the mentioned terminations, we also considered those that may be present on the surface *via* the HF etching or by contact with the water medium, *i.e.*, −F, −O, −OH, and −H terminations. Although these terminations often fail to meet the energetic criteria for Sc_2_C and Y_2_C, they may still be present on the surface and consequently hinder the photocatalytic performance.

As thoroughly established in a recent work,^24^ among the studied MXenes, all of them favored ABC stacking over ABA, while the termination position varied depending on the T_*x*_ atom. For Zr_2_CT_2_ MXenes, all the structures were more energetically stable when the termination occupied the H_M_ hollow. For Sc_2_CT_2_ and Y_2_CT_2_, the halide terminations (−F, −Cl, −Br, −I), −OH, and −H also preferred the H_M_ positions, while for chalcogen terminations (−O, −S, −Se) H_MX_ was the most favorable hollow site. The most stable structure for each case is the one used for the following calculations.

### 2.2 Methods

The Vienna *ab initio* simulation package (VASP) code was employed to carry out electronic structure calculations^45^ within the DFT framework.^46,47^ The Perdew–Burke–Ernzerhof (PBE)^48^ exchange-correlation (*xc*) functional, based on the generalized gradient approximation (GGA),^49^ was used in all MXenes geometry optimizations, with Grimme’s D3 correction added to include dispersive forces.^50^ Still, to get accurate results for the bandgap and excited state energies, the PBE0 hybrid *xc* was applied.^51,52^ The optimization of the electronic structure was performed with a threshold of 10^–6^ eV for the self-consistent field steps, while the geometrical optimizations were considered converged when atomic forces were lower than 0.01 eV·Å^–1^. During the optimizations, all atomic positions and cell parameters were allowed to relax. To represent the core electrons and their interaction with the valence electrons, projector augmented wave (PAW) pseudopotentials were used,^53^ while a planewave basis set with an optimal kinetic energy cutoff of 415 eV was chosen to describe the valence electron density.^54^ For the Brillouin zone integration, optimal **Γ**-centered 7 × 7 × 1 Monkhorst–Pack **k**-point grids were employed.^55^ Vibrational frequencies for MXenes were calculated using finite displacements of 0.02 Å on the termination atoms. Spin-polarized calculations were carried out for each system, though the ground state of the studied terminated MXenes is a closed-shell singlet, without any magnetization, as known for other terminated MXenes.^56^ To model the singlet photoexcited state, the electronic state is assessed using a triplet (T_1_) spin configuration with two unpaired electrons and a total magnetization of two. This approximation enables structural optimization on the excited state potential energy surface while introducing only a minor error in the energy.^57^ The energy difference between the triplet and the open-shell singlet states mainly arises from the exchange interaction term between a spatially separated electron and hole, resulting in a relatively small value. This approximation has been successfully applied in the study of the luminescence spectra, the character, localization, and diffusion of the photogenerated electrons and holes,^58^ and in the study of the mechanism of photocatalytic reactions.^59^ While the GW with the Bethe–Salpeter equations (GW+BSE) approach is the gold standard for modeling excitons in semiconductors,^60,61^ applying it to calculations across such a vast compositional space is computationally too expensive. Instead, we rely on our previous rigorous benchmarking of these specific MXenes,^32^ which demonstrated that bandgaps predicted by the PBE0 hybrid functional correlate well with those acquired by GW calculations. By effectively mitigating self-interaction errors, PBE0 provides a robust and computationally viable alternative for accurately modeling the T_1_ state. It should be noted that all calculations were performed in vacuum, without the inclusion of implicit or explicit solvation models. Following the established methodology of reference works computing ground-state Pourbaix diagrams for MXenes,^22,38,40^ the gas-phase approach allowed us to isolate the intrinsic thermodynamic shifts induced by photoexcitation from solvent-related variables, although including solvation effects represents an important next step for refining these models.

### 2.3 Pourbaix Diagrams

Similar to the conventional pressure–temperature phase diagrams, Pourbaix diagrams identify the thermodynamically most stable phase of a material under specific working conditions. However, instead of pressure and temperature, Pourbaix diagrams are defined by pH and the applied electrochemical potential, *U*, while pressure and temperature are typically kept constant at standard conditions (25 °C and 1 bar). These Pourbaix diagrams are created by first selecting a reference structure from the different ones studied. In our context, these would be the pristine MXene surfaces, *i.e.*, Zr_2_C, Sc_2_C, and Y_2_C, in their ground and excited triplet states. Then, the possible adsorbed terminations on the surface are described by a specific set of reactions, see Table 1.

**Table 1:** Set of Reactions to Describe the Adsorption Process of the Different Terminations Onto MXenes^a^

T_*x*_	Reaction	Computational Electrode
O	M_2_X + 2H_2_O → M_2_XO_2_ + 2H_2_	M_2_X + 2H_2_O → M_2_XO_2_ + 4H^+^ + 4*e* ^–^
OH	M_2_X + 2H_2_O → M_2_X(OH)_2_ + H_2_	M_2_X + 2H_2_O → M_2_X(OH) _2_ + 2H^+^ + 2*e* ^–^
H	M_2_X + H_2_ → M_2_XH_2_	M_2_X + 2H^+^ + 2*e* ^–^ → M_2_XH_2_
Y (F, Cl, Br, I)	M_2_X + Y_2_ → M_2_XY_2_ (*U*_Y_)	M_2_X + 2Y^–^ → M_2_XY_2_ + 2*e* ^–^ (*U*_Y_)
Z (S, Se)	M_2_X + 2Z → M_2_XZ_2_ (*U*_Z_)	M_2_X + 2Z^2–^ → M_2_XZ_2_ + 4*e* ^–^ (*U*_Z_)

a For each termination, the representation of the reaction being used and the corresponding computational electrode is also given.

The free energy change associated with these reactions can be initially expressed without considering the effects of the pH nor *U* (assuming both are zero). In that case, the expression is as follows:

1 $$\Delta G\left(0,0\right)=\Delta E+\Delta E_{\mathrm{ZPE}}-T\Delta S$$

where *T* is the temperature, Δ*E* is the total energy change, Δ*E_ZPE_* refers to the zero point energy (ZPE) difference, and Δ*S* is the variation in entropy, all calculated between the products and the reactants. For example, in a general reaction of the form *v*_*R*_1__ *R*_1_ + *v*_*R*_2__ *R*_2_+ ... → *v*_*P*_1__ *P*_1_ + *v*_*P*_2__ *P*_2_ + ..., where *R* and *P* are the reactants and products, respectively, and *v* their corresponding stoichiometric coefficients, the total energy change would be calculated as follows:

2 $$\Delta E=\underset{P}{\sum }v_{P}E_{P}-\underset{R}{\sum }v_{R}E_{R}$$

Then, Δ*E_ZPE_* and Δ*S* can be computed in a similar fashion. For a given system, the ZPE and the vibrational contribution to the entropy, *S*_vib_, can be computed from the vibrational frequencies, *ν*, of the different normal modes of vibration (NMV):

3 $$E_{\mathrm{ZPE}}=\frac{1}{2}\overset{\mathrm{NMV}}{\underset{i}{\sum }}h\nu_{i}$$

4 $$S_{\mathrm{vib}}=k_{B}N_{A}\overset{\mathrm{NMV}}{\underset{i}{\sum }}\left[\frac{\frac{h\nu_{i}}{k_{B}T}}{e^{h\nu_{i}/k_{B}T}-1}-\ln\left(1-e^{-\frac{h\nu_{i}}{k_{B}T}}\right)\right]$$

where *h*, *k*_B_, and *N*_A_, correspond to Planck’s constant, Boltzmann’s constant, and Avogadro’s number, respectively. For the gaseous free molecules, the entropy has been obtained from thermodynamic tables.^62,63^ While these can be derived from DFT *via* the rigid rotor-harmonic oscillator (RRHO) approximation, our benchmark tests for H_2_O, H_2_, O_2_, and F_2_ indicated that DFT-calculated entropies align with experimental tables within a minimal error margin—lower by −0.2% in average. Given this high degree of agreement, experimental values were preferred to provide the most accurate description of the gas-phase entropic contribution in the Pourbaix diagrams. For the MXene-based species (pristine or terminated), the entropy is assumed to be dominated by the vibrational component, and, in addition, we suppose that the only contribution to the vibrations is related to the adsorbates, as extensively used in past studies.^22,38,64^ This means that for the terminated MXenes, we assume that the ZPE and entropy solely originate from the adsorbed terminations, while for pristine MXenes, both are considered negligible.

Over the free energy change expressed in eq 1, one can introduce the potential, relative to the standard hydrogen electrode (SHE), *U*, and the pH, by using the widely employed computational hydrogen electrode (CHE).^65^ By analyzing the reaction used to form a particular terminated structure from the reference pristine structure, we can determine the number of protons, $v_{H^{+}}$ , and electrons, $v_{e^{-}}$ , involved in the process. This information is essential for constructing a Pourbaix diagram, as the effects of pH and *U* are not directly included in DFT calculations but are instead incorporated *a posteriori* as a thermodynamic correction, resulting in the following expression:

5 $$\Delta G\left(\mathrm{pH},U\right)=\Delta G\left(0,0\right)-v_{H^{+}}k_{B}T\;\ln\left(10\right)\;pH-v_{e^{-}}eU$$

where *e* is the elementary charge of an electron. Similar to the CHE, we can use other computational electrodes to include the reference for different types of terminations.^22,38^ For instance, for halide terminations (Y = F, Cl, Br, I), the corresponding reversible Y_2_/Y^–^ redox pair, given by eq 6, is considered, with a redox potential, *U*_Y_, of 2.87, 1.36, 1.07, and 0.54 V for F, Cl, Br, and I, respectively,^62,66^ whereas for chalcogen terminations (Z = S, Se), the Z/Z^2–^ redox pair, given by eq 7, was selected, as done before for S-terminated MXenes,^67^ with the respective potential values, *U*_Z_, of −0.48 and −0.67 V versus SHE at standard conditions.^62,66^

6 $$Y_{2}+2e^{-}⇌2Y^{-}\left(U_{Y}\right)$$

7 $$Z+2e^{-}⇌Z^{2-}\left(U_{Z}\right)$$

These computational electrodes can be easily implemented into eq 5 by adding an additional term indicating the stoichiometric coefficient of the termination anions, *v*_T_, combined with the equilibrium potential of the reaction, *U*_T_. Then, the equation becomes

8 $$\Delta G\left(\mathrm{pH},U\right)=\Delta G\left(0,0\right)-v_{H^{+}}k_{B}T\;\ln\left(10\right)pH-v_{e^{-}}eU-v_{T}eU_{T}$$

With this, we have the full representation of stability, in terms of Δ*G*, as a function of pH and *U* for the different MXenes and their terminations. Comparing the different Δ*G* values, the regions of thermodynamic stability corresponding to the most stable terminated phases can be mapped, resulting in the built Pourbaix Diagram. The diagrams in this work were generated using an in-house-developed Python script designed for systematic pH-*U* mapping, available for its use.^68^ Finally, to investigate the effect of photoexcitation on surface stability, Pourbaix diagrams were also constructed in the excited state. For this purpose, the total energies and vibrational properties were calculated in the excited triplet state, using both PBE and PBE0 functionals for a more reliable representation of the bandgap and energies.

## 3 Results

### 3.1 Ground-State Pourbaix Diagrams

Once the most stable structures are identified for each terminated MXene (see the Structural Models section), the ground and excited state Pourbaix diagrams can be obtained through DFT calculations, as mentioned in the computational details. These can be found in Figure 2, as computed at the PBE level. Generally, at lower *U*, the system is fully reduced, and the H atoms are the most favorable terminations at any pH. As the external potential increases, −F and −OH terminated regions start to appear, except for the ground state Zr_2_C MXene, which transitions almost directly to the O-terminated case. Finally, at higher positive *U*, all MXenes become fully oxidized and predominantly covered with −O terminations, except for the group III MXenes in their ground state, which exhibit a narrow region at low pH where S atoms are the preferred surface termination. In all cases, across the considered pH and *U* conditions, the most stable phases correspond to a terminated surface. This clearly indicates that obtaining and maintaining pristine, unfunctionalized MXenes is essentially impossible under working conditions; under aqueous conditions, or during the synthesis process, where reactions can introduce surface terminations, termination groups are likely to attach to the MXene surface.

**Figure 2:**
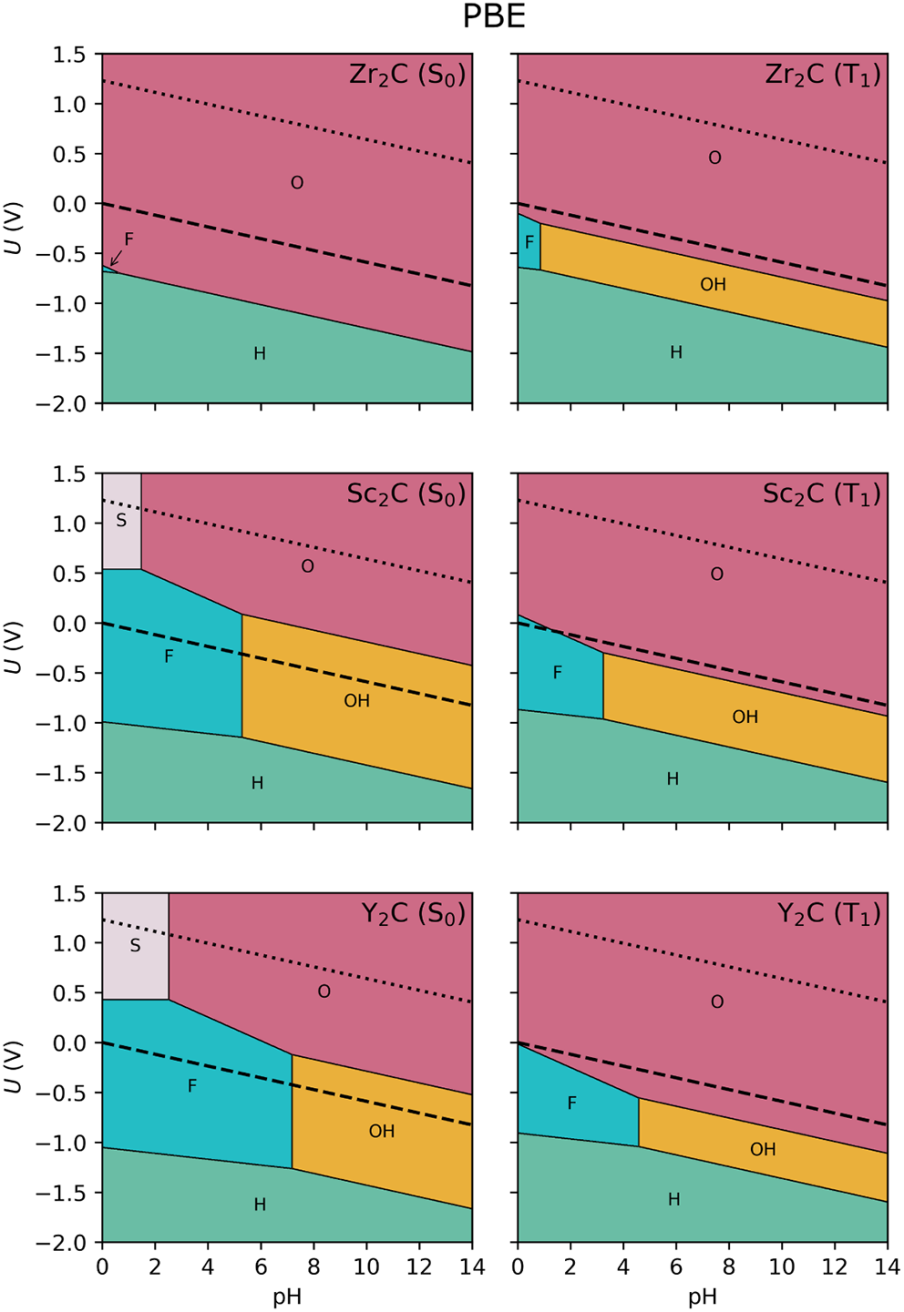
Surface Pourbaix diagram at the PBE level for the ground (S_0_, left) and excited (T_1_, right) states for the considered Zr_2_C, Sc_2_C and Y_2_C MXenes. The dashed black line indicates the HER equilibrium potential (*U* = 0 V *vs.* SHE), and the dotted one indicates the OER potential (*U* = 1.23 V *vs*. SHE).

For the Sc_2_C and Y_2_C cases, the F- and OH-terminated surfaces are quite dominant in the S_0_ Pourbaix diagram, encompassing entirely the HER redox potential line at *U* = 0 V. This suggests that, at least within the PBE framework, under HER operando conditions, −F and −OH will be the most favored surface terminations. On the contrary, when increasing *U* toward OER conditions (*U* = 1.23 V), −S and −O terminations are preferred over −OH and halogens. In contrast, the Zr_2_C MXene maintains the −O termination for a wider range (*U* > −0.5 V), making it the most stable phase under both HER and OER conditions. Note that, since in the presently constructed Pourbaix diagrams we used only fully terminated cases, we are not accounting for mixed situations. Such mixed terminations are actually frequent and found in the vicinities between two fully terminated cases.^22^ For instance, the change from −OH to −O termination is not abrupt, and in between different states with different OH/O ratios will exist, yet here are not accounted for.

### 3.2 Excited-State Pourbaix Diagrams

By examining the diagrams in the excited T_1_ state, where the photoelectrocatalytic reaction is expected to occur, we observe significant changes in the relative stabilities of different surface terminations (see Figure 2). Although the overall shapes of the stability regions are preserved, notable differences emerge. For instance, the S-terminated domains observed in the ground-state diagrams for Sc_2_C and Y_2_C disappear entirely in the excited state, while the F- and OH-terminated regions shrink considerably. As a result, under HER conditions, the −O termination becomes thermodynamically favored. This behavior can be rationalized in terms of the relative energetic stability of the excited states on the terminated MXenes. Table S1 of the Supporting Information (SI) provides the values for Δ*G*(0,0), which already include the termination contribution from eq 8 —the $-v_{T}eU_{T}$ term. These values allow for a comparison of the relative stabilities of different surface terminations for both ground S_0_ and excited T_1_ states. For example, the excited state of the S-terminated phase is substantially less stable than that of the O-terminated phase, lying at higher energies relative to the corresponding pristine MXene. Consequently, upon photoexcitation, the free-energy difference reverses: The narrow S-terminated region found in the ground state is replaced by a more stable O-terminated surface. Quantitatively, in the S_0_ ground state, the Δ*G*(0,0) difference between S- and O-terminated phases is 0.35 eV for Sc_2_C (0.59 eV for Y_2_C), favoring the S-terminated MXene. In the excited T_1_ state, however, the preference is reversed, with the O-terminated phase being more stable by 0.80 eV for Sc_2_C (0.82 eV for Y_2_C). A similar trend is observed for the −F and −OH terminations, which become less stable than −O and −H in the excited T_1_ state, thereby reducing their stability domains in the corresponding Pourbaix diagrams.

On the contrary, for Zr_2_C, T_1_ shows narrow stability regions for F- and OH-terminated surfaces, while −O termination remains the most favorable under both HER and OER conditions. This behavior is again linked to the relative stability of the excited states. For Zr_2_CO_2_, the T_1_ excited state is relatively less stable than Zr_2_CF_2_ and Zr_2_C­(OH)_2_. This may be attributed to the significantly larger bandgap of the O-terminated phase,^32^ which shifts its excited state to higher energies, thereby destabilizing it relative to the F- and OH-terminated counterparts.

From this analysis, it is clear that the terminations typically resulting from aqueous HF synthesis (−F, −O, −OH, and −H) dominate the stability regions in both the ground and excited states. This is important, especially for Sc_2_C and Y_2_C MXenes, where halide and chalcogen terminations show no stability domains, with the sole exception of the mentioned small region of −S. For Zr_2_C, the O-terminated surface (Zr_2_CO_2_) is of primary interest due to its superior photocatalytic properties for water splitting.^44^ Luckily, this termination is also the most stable configuration across most pH and potential ranges, including the regions corresponding to HER and OER conditions. However, the situation is different for Sc_2_C and Y_2_C MXenes, where the more promising candidates for photocatalytic water splitting are when terminated with T_*x*_ = Cl, Br, S, and Se.^24^ In these cases, HF-based synthesis may hinder photocatalytic performance by favoring other terminations, and alternative synthesis strategies, like electrochemical and Lewis acid molten salt etching, that provide better control over the surface termination are better alternatives.^30,31,69^

Summarizing, the physical origins of these stability trends can be traced to the interplay of bond strength, electronic structure, and excitation energy. The high electropositivity of early transition metals drives the formation of strong bonds with electronegative species, explaining the thermodynamic dominance of −F, −O and −OH across the diagrams. However, the specific behavior of each MXene depends heavily on its metal valency. Because Zr is a Group IV metal, it possesses the optimal number of valence electrons to fully coordinate with O, forming a stable structure, that Group III metals like Sc and Y cannot replicate, leading to smaller O-terminated regions. Finally, the rearrangements in the T_1_ state are driven by the excitation penalty; since the energy scales with the bandgap, surface terminations yielding wider bandgaps experience greater relative destabilization under light.

### 3.3 PBE *vs.* PBE0 Functionals

It is well known that the PBE functional underestimates the bandgap of semiconductor materials, and it does the same for the energy difference between the ground and the lowest excited state. To improve the accuracy of these predictions and assess whether it affects the shape of the Pourbaix diagram, the energies and vibrational frequencies were recomputed by applying the PBE0 hybrid functional, resulting in the Pourbaix diagrams of Figure 3. The general shape of the stability regions remains consistent between the two levels of theory, suggesting that the underestimation of the bandgap by PBE does not qualitatively alter the predicted surface terminations. Nevertheless, some noticeable differences emerge. For example, the stability region of F-terminated surfaces expands under PBE0, highlighting a slightly stronger thermodynamic preference for fluorine functionalization. In addition, a small stability domain for −Se termination appears near pH ∼ 0 for Sc_2_C in the T_1_ excited state, a feature absent in the PBE-based diagrams. A particularly relevant change is observed at the Zr_2_C HER potential in the excited state; with PBE0, the expansion of the F- and OH-terminated domains leads to the HER line being encompassed by these terminations rather than by the desired −O termination. Nevertheless, the HER line lies very close to the O-terminated domain, indicating that small shifts in free energy could favor −O termination under realistic conditions. Overall, this illustrates that PBE can capture the essential features of the Pourbaix stability maps, and hybrid *xc* functionals such as PBE0 provide an improved quantitative accuracy and may reveal secondary trends, particularly in cases where competing terminations have comparable free energies.

**Figure 3:**
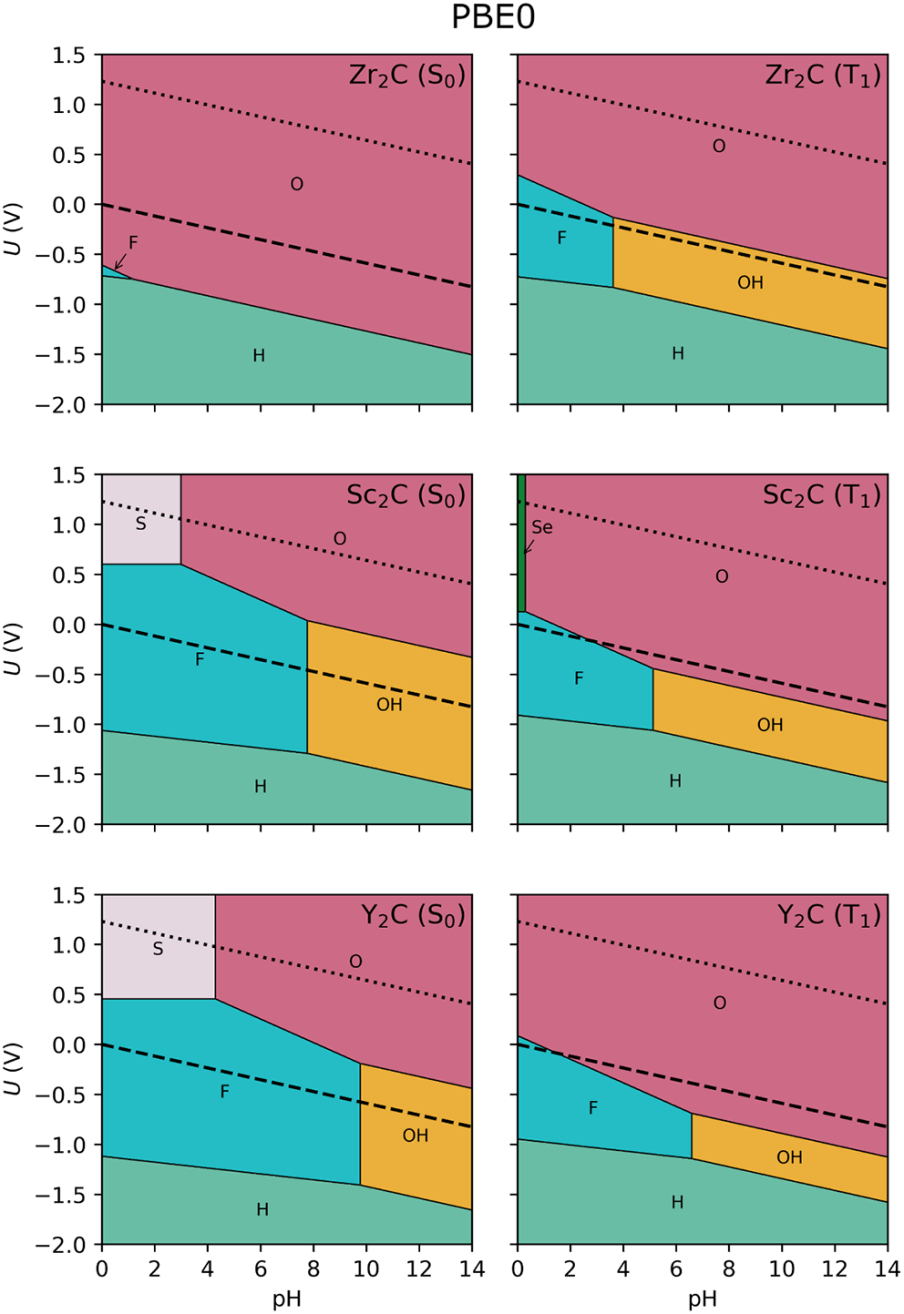
Surface Pourbaix diagram at PBE0 level for the ground (S_0_, left) and excited (T_1_, right) states for the considered Zr_2_C, Sc_2_C and Y_2_C MXenes. The dashed black line indicates the HER equilibrium potential (*U* = 0 V *vs.* SHE), and the dotted one indicates the OER potential (*U* = 1.23 V *vs.* SHE).

One thing to notice is that, among the different surface groups considered, −F terminations display a distinct behavior compared to O-, H-, or OH-functionalized surfaces. In the Pourbaix diagrams, the stability domains of F-terminated MXenes are confined to low pH values. This can be rationalized by the fact that the free energies of H-, O-, and OH-terminated surfaces are strongly pH-dependent, whereas the free energy of −F remains essentially insensitive to pH. This dependency can be seen in the chemical equations of Table 1 and the corresponding *v*_H+_ coefficients gathered in Table S1 of the SI. In the adsorption process of halide and chalcogen terminations, there are no H^+^ or H_2_ species involved, and thus $v_{H^{+}}=0$ , removing the pH term in eq 8 and making the process only *U*-dependent. As a result, fluorine can compete effectively under acidic conditions, but its relative stability diminishes quickly as pH increases, where −H, −O, and −OH become thermodynamically favored. The preference for −F termination over other halides can be understood in terms of relative adsorption free energies. Fluorine atoms bind more strongly to the MXene surface, which is reflected in the Δ*G*(0,0) values reported in Table S1 of the SI. As expected from periodic trends, this stability decreases down the halogen group, following the order *F* > Cl > Br > I.

To gain further insights into the relative stability of the different terminations, the free energy as a function of pH at fixed potential and as a function of potential at fixed pH were also analyzed, see Figures S1–S4 of the SI. These plots represent the underlying thermodynamic trends that determine the Pourbaix stability regions, allowing us to visualize directly how Δ*G* for each termination evolves with the electrochemical environment. For instance, at fixed *U* (see, for example, the *U* = 0 V case of Figure S3 of the SI) the free energies of O- and OH-terminated surfaces decrease with increasing pH, whereas for −H termination, it increases, while halogen, −S and −Se terminations remain essentially flat, emphasizing their pH-independent behavior as discussed above. These profiles also highlight the relative ordering of stability between different terminations. This is particularly important in cases where the free energy differences are small enough to allow for the coexistence of multiple surface groups. For example, the narrow Se-terminated domain observed in the excited state of Sc_2_C (Figure 3) corresponds to a near-degeneracy in free energy with the O-terminated surface at pH = 0 (*cf.* Figure S1 of the SI) and at small pH for *U* = 1.23 V (*cf.* Figure S4 of the SI). Similarly, at pH = 7 (see Figure S2 of the SI), the F- and OH-terminated phases of Sc_2_C and Y_2_C are nearly indistinguishable in energy, both in the S_0_ ground and T_1_ excited states, suggesting that these terminations may compete or even coexist. With this in mind, these analyses reinforce that while the present study focuses on the effect of photoexcitation on MXene compositional stability, more realistic models should also account for possible mixtures or coexistence of surface terminations, as emphasized in previous works.^22,38^

### 3.4 Stability of Alternative Terminations

Finally, to assess the stability of terminated MXenes beyond the typical aqueous- and HF-derived terminations, we analyzed the Pourbaix diagrams of Sc_2_C and Y_2_C while excluding −F, −H, −OH, and −O. These two cases are particularly relevant because HF-derived terminations tend to passivate the photoactivity of the materials. By removing them from consideration, we can better evaluate the intrinsic stability of alternative surface terminations. First, we analyzed the PBE0 diagrams excluding −F, representing an aqueous but F-free environment. The results, available in Figure S5 of the SI, closely resemble those in Figure 3, with the OH-terminated region now expanding to occupy the area previously dominated by −F terminations. In the ground state, this also leads to an extension of the S- and H-terminated phases toward lower and higher *U*, respectively. For Y_2_C in the ground state, a small stability region favoring −Cl termination appears near pH ∼ 0. This behavior changes in the T_1_ excited state, where a very narrow stability region corresponding to the Br-terminated phase emerges slightly above pH = 0. These results indicate that under low pH aqueous conditions, Cl- and Br-terminated Y_2_C surfaces, both previously reported as photoactive for water splitting,^24^ are thermodynamically attainable.

The diagrams without −F, −H, −OH, and −O have also been investigated at the PBE0 level, see Figure 4. In the ground state, −Cl and −S are identified as the most stable terminations. Upon photoexcitation, however, the stability landscape changes: In both systems, −S is replaced by Se, while in Y_2_C the −Cl termination is further substituted by Br, indicating a relative stabilization of heavier chalcogen and halogen terminations in the excited state. Notably, both MXenes display a narrow region at highly reducing potentials (*U* < −1.5 V) where the pristine, unfunctionalized surface becomes thermodynamically preferred.

**Figure 4:**
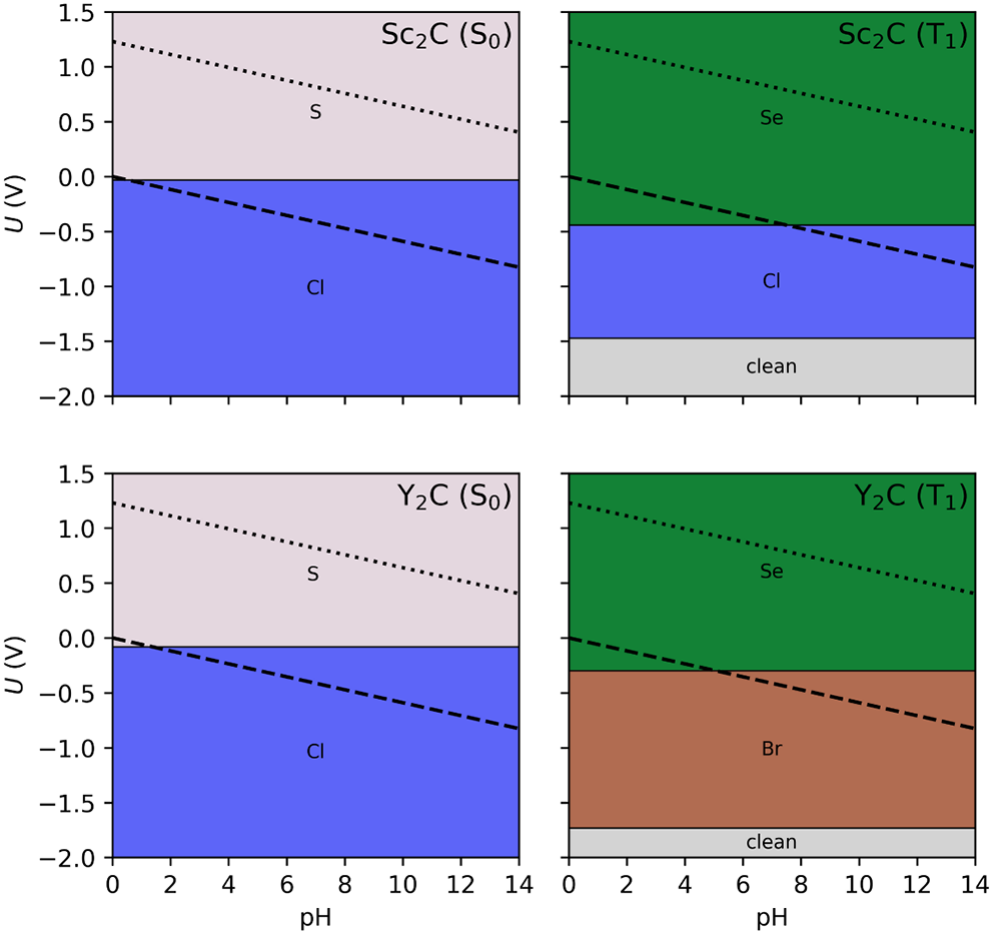
Surface Pourbaix diagram at the PBE0 level for the ground (S_0_, left) and excited (T_1_, right) states for the considered Sc_2_C and Y_2_C MXenes, without considering terminations derived from HF synthesis (−H, −OH, −O, −F). The dashed black line indicates the HER equilibrium potential (*U* = 0 V *vs.* SHE), and the dotted one indicates the OER potential (*U* = 1.23 V *vs.* SHE).

## 4 Conclusions

In this work, a novel application of Pourbaix diagrams in the excited state is presented to study the surface stability of terminated MXenes under photoelectrochemical conditions. Here, we focus on Sc_2_C, Y_2_C and Zr_2_C, a group of MXenes previously reported as promising photocatalysts for the water splitting reaction, but whose stability under realistic photoelectrochemical treatment has not yet been properly assessed. By combining ground- and excited-state DFT calculations, it is shown that the photoexcitation can significantly alter the surface termination stability, shifting the thermodynamically favored phases and directly influencing the photocatalytic performance. This underscores that evaluating thermodynamic stability solely in the ground state is insufficient for predicting the behavior of photocatalytic materials under operating conditions.

The results reveal that terminations typically resulting from aqueous HF-based processing, such as −F, −O, −OH, and −H, dominate the stability regions in both ground and excited states. However, photoexcitation reduces the stability of some of these terminations, such as −F and −OH in Sc_2_C and Y_2_C, and, in some cases, introduces new stability regions, e.g., −O termination under HER conditions. For Zr_2_C, −O termination remains predominant under HER and OER regimes, although narrow regions for −F and −OH terminations emerge in the excited state. Importantly, we also find that some terminations can be nearly degenerate in free energy, meaning that multiple phases with different mixture ratios could coexist under certain conditions. This competition is not always directly visible in the Pourbaix diagram but becomes apparent when analyzing the underlying free-energy profiles, and it may play a key role in surface reactivity.^22^

These findings also emphasize the role of synthesis routes in determining photocatalytic performance. While HF-etching-derived terminations dominate under most environments, our results suggest that halide and chalcogen terminations (−Cl, −Br, −S, −Se) could provide more favorable surfaces for water splitting, especially in Sc_2_C and Y_2_C. Achieving these terminations may therefore require alternative approaches, such as water- and F-free techniques, electrochemical etching, or Lewis acid molten salt methods, that allow finer control over the final surface composition. By comparing PBE and PBE0 functionals, it is shown that PBE can capture the essential features of the Pourbaix stability maps, while hybrid functionals such as PBE0 provide improved quantitative accuracy and may reveal secondary trends, particularly in cases where competing terminations have comparable free energies.

From a practical perspective, the present study shows how excited-state Pourbaix diagrams can be gained, providing a powerful tool to predict surface stability under realistic operational conditions, which is essential for designing and selecting MXene-based photocatalysts. In practice, for Sc_2_C and Y_2_C MXenes, tailoring synthesis routes to stabilize non-HF-derived terminations could be crucial to optimize their activity. For Zr_2_C, −O termination emerges as both the most stable and photocatalytically promising configuration across most pH and *U* ranges.

## Supplementary Material



## Data Availability

The data and Python code used to create the diagrams and plots are available at https://github.com/diegonti/pourbaix_plot. Any additional data is available from the corresponding authors upon reasonable request.

## Notes

Contains a data table for the calculated Δ*G*(0,0) values, Δ*G* plots as a function of pH (at fixed *U*) and *U* (at fixed pH), and the Pourbaix diagram without considering −F terminations (PDF)
