# Supplementary material for: MXenes Surface Termination under Photoexcitation: Insights from Excited-State Pourbaix Diagrams
Source: ACS Appl Mater Interfaces. 2026 Mar 10;18(11):16573–82. doi: 10.1021/acsami.6c00715 (PMC13022819; doi:10.1021/acsami.6c00715)

MXenes Surface Termination under Photoexcitation: Insights from Excited-State Pourbaix Diagrams

Diego Ontiveros, Francesc Viñes, and Carmen Sousa*

Departament de Ciència de Materials i Química Física & Institut de Química Teòrica i Computacional (IQTCUB), Universitat de Barcelona, c/ Martí i Franquès 1-11, 08028, Barcelona, Spain

* Corresponding author: c.sousa@ub.edu

Table S1. Calculated $\Delta G(0,0)$ energies, in eV, adding also the termination term ($-v_T e U_T$, see definition in Equation 8 of the main text) at PBE, $\Delta G^{PBE}(0,0)$, and PBE0, $\Delta G^{PBE0}(0,0)$, density functional exchange-correlation levels, and stoichiometric coefficients v_{H^+}, v_{e^-}, and v_T for the considered terminated MXenes in the ground singlet, S_0, and excited triplet, T_1, state.

MXene	State	T_x	$\Delta G^{PBE}(0,0)$	$\Delta G^{PBE0}(0,0)$	v_{H^+}	v_{e^-}	v_T
Zr_2C	S_0	**clean**	0.00	0.00	0	0	0
		**O**	-6.41	-6.67	4	4	0
		**OH**	-4.90	-4.93	2	2	0
		**H**	-2.45	-2.59	-2	-2	0
		**F**	-5.17	-5.45	0	2	-2
		**Cl**	-4.67	-4.95	0	2	-2
		**Br**	-4.35	-4.67	0	2	-2
		**I**	-3.79	-3.93	0	2	-2
	T_1	**clean**	0.00	0.00	0	0	0
		**O**	-3.99	-3.68	4	4	0
		**OH**	-3.69	-3.84	2	2	0
		**H**	-1.23	-1.37	-2	-2	0
		**F**	-3.80	-4.27	0	2	-2
		**Cl**	-3.44	-3.78	0	2	-2
		**Br**	-3.16	-3.55	0	2	-2
		**I**	-2.71	-2.96	0	2	-2
Sc_2C	S_0	**clean**	0.00	0.00	0	0	0
		**O**	-4.60	-4.97	4	4	0
		**OH**	-5.40	-5.96	2	2	0
		**H**	-2.06	-2.63	-2	-2	0
		**F**	-6.02	-6.87	0	2	-2
		**Cl**	-4.86	-5.61	0	2	-2
		**Br**	-4.44	-5.20	0	2	-2
		**I**	-3.74	-4.35	0	2	-2
		**S**	-4.95	-5.67	0	4	-2
		**Se**	-4.53	-5.31	0	4	-2
	T_1	**clean**	0.00	0.00	0	0	0
		**O**	-3.72	-3.76	4	4	0
		**OH**	-3.50	-3.48	2	2	0
		**H**	-0.42	-0.45	-2	-2	0
		**F**	-3.89	-4.08	0	2	-2
		**Cl**	-2.84	-2.95	0	2	-2
		**Br**	-2.47	-2.39	0	2	-2
		**I**	-2.23	-2.42	0	2	-2
		**S**	-2.92	-2.89	0	4	-2
		**Se**	-3.61	-3.83	0	4	-2
		**clean**	0.00	0.00	0	0	0
		**O**	-4.71	-4.97	4	4	0
		**OH**	-5.32	-5.74	2	2	0
		**H**	-1.96	-2.41	-2	-2	0

	**S_0**	**F**	-6.16	-6.89	0	2	-2
		**Cl**	-5.17	-5.82	0	2	-2
		**Br**	-4.90	-5.60	0	2	-2
		**I**	-4.49	-5.08	0	2	-2
		**S**	-5.31	-5.98	0	4	-2
**Y_2C**		**Se**	-4.92	-5.69	0	4	-2
	**T_1**	**clean**	0.00	0.00	0	0	0
		**O**	-4.09	-4.07	4	4	0
		**OH**	-3.52	-3.46	2	2	0
		**H**	-0.43	-0.45	-2	-2	0
		**F**	-4.06	-4.24	0	2	-2
		**Cl**	-3.23	-3.37	0	2	-2
		**Br**	-3.01	-3.47	0	2	-2
		**I**	-2.89	-3.28	0	2	-2
		**S**	-3.27	-3.42	0	4	-2
		**Se**	-3.89	-4.06	0	4	-2

Figure S1. Relative stability, in terms of ΔG, in eV, of the different terminated MXene phases as a function of the external potential, U, in V, at a fixed pH = 0 and at PBE0 level. The colored shaded regions indicate the most stable termination in each potential window.

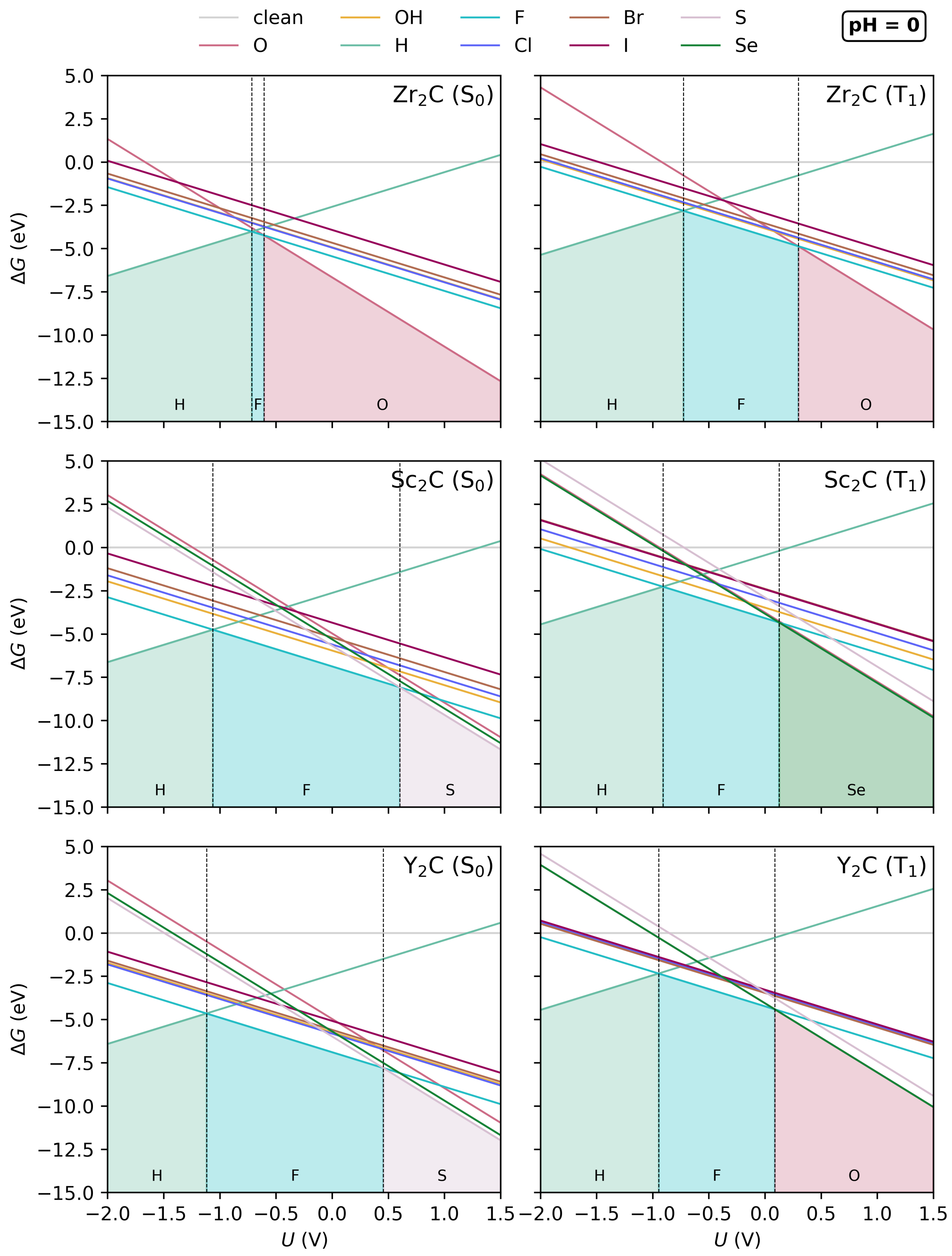

Figure S2. Relative stability, in terms of the ΔG, in eV, of the different terminated MXene phases as a function of the external potential, U, in V, at a fixed pH = 7 and at PBE0 level. The colored shaded regions indicate the most stable termination in each potential window.

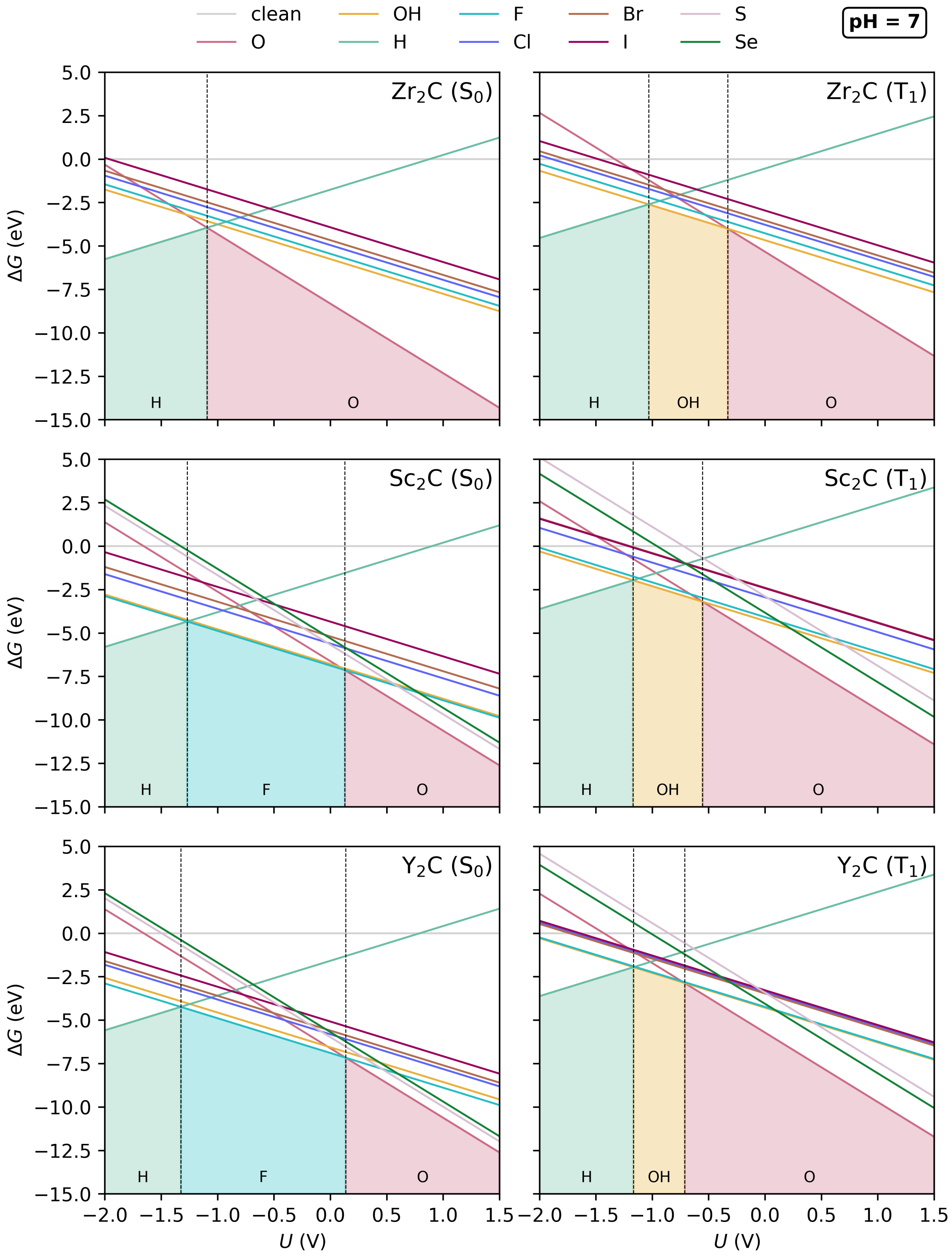

Figure S3. Relative stability, in terms of the ΔG, in eV, of the different terminated MXene phases as a function of the pH, at a fixed U = 0 V and at PBE0 level. The colored shaded regions indicate the most stable termination in each pH window.

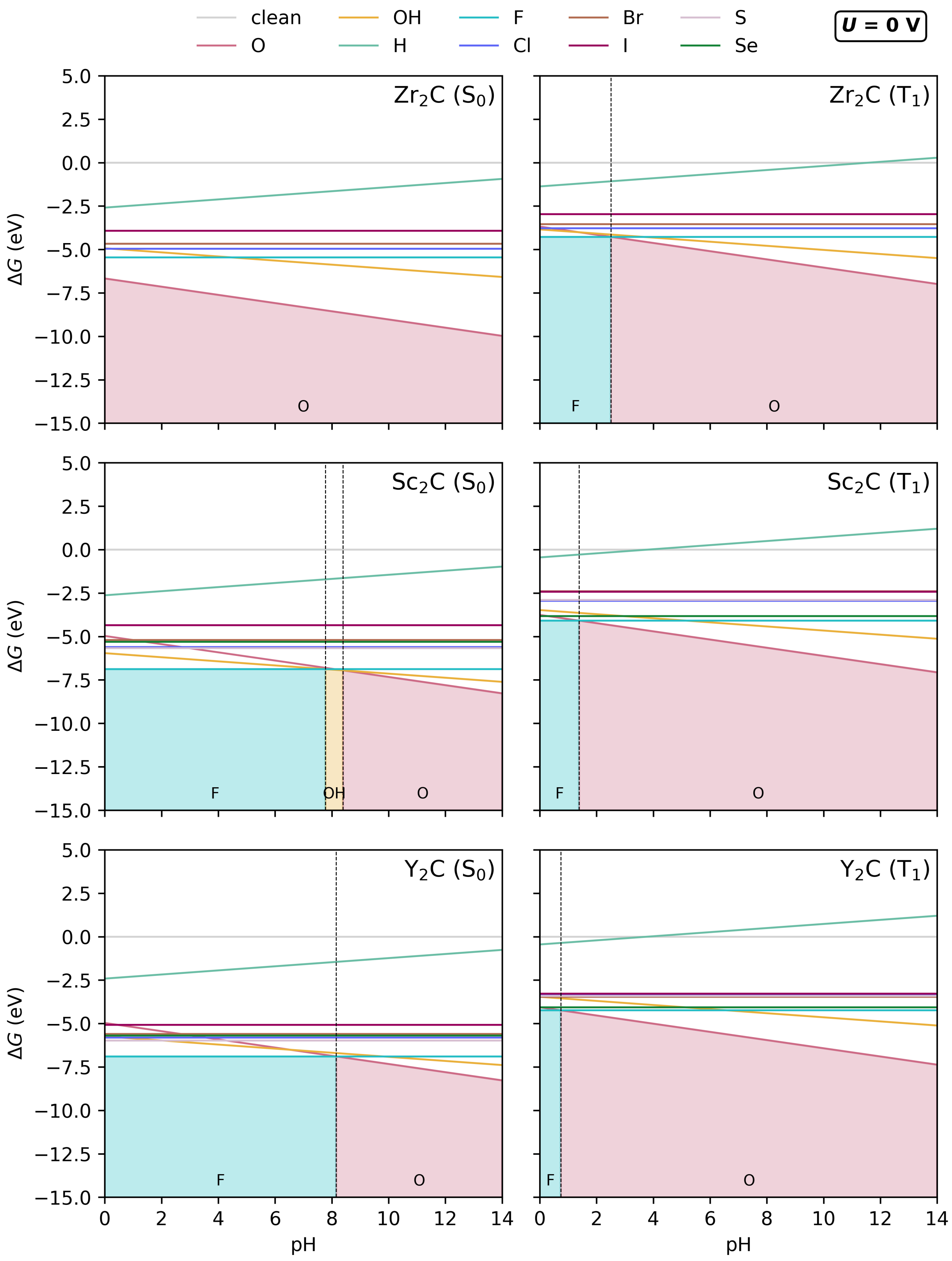

Figure S4. Relative stability, in terms of the *ΔG*, in eV, of the different terminated MXene phases as a function of the pH, at a fixed U = 1.23 V and PBE0 level. The colored shaded regions indicate the most stable termination in each pH window.

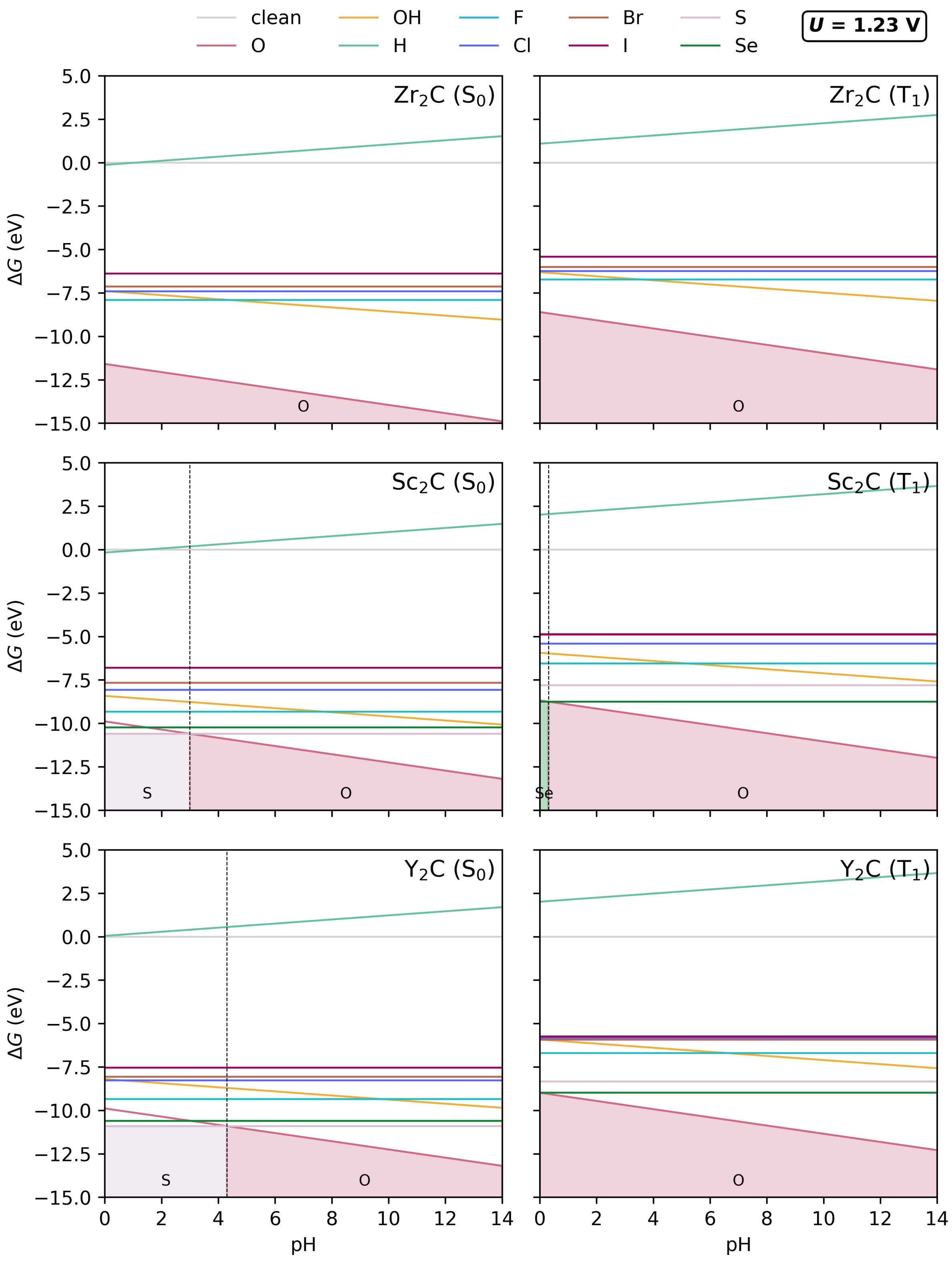

Figure S5. Surface Pourbaix diagram at PBE0 level for the ground (S_0, left) and excited (T_1, right) states for the Sc_2C and Y_2C MXenes, without considering –F termination. The inset in Y_2C (T_1) shows an expanded region around pH = 0, to showcase the Br-terminated area. The dashed black line indicates the HER equilibrium potential (U = 0 V vs. SHE) and the dotted one the OER potential (U = 1.23 V vs. SHE).

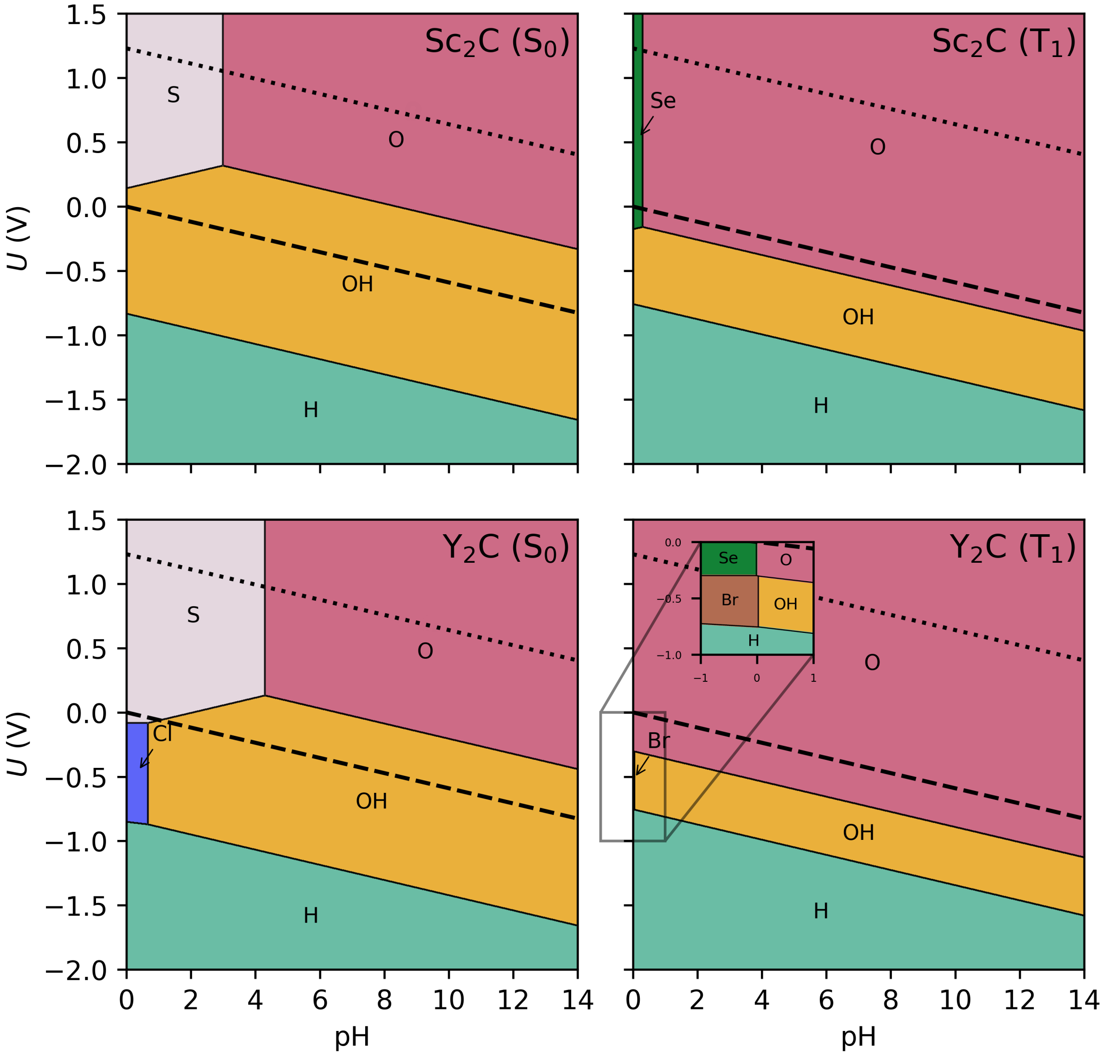

Supplement: Supplementary file 1 [file am6c00715_si_001.pdf]
